# *The Organon* and Materia Medica Club of the Bay Cities of California

**Published:** 1898-06

**Authors:** Guy E. Manning

**Affiliations:** Secretary


### *THE ORGANON* AND MATERIA MEDICA CLUB OF THE BAY CITIES OF CALIFORNIA.

The Bay City *Organon* and Materia Medica Club was called to order by the President October 1st, 1897, at 8.30 P. M.

The members present were Drs. J. M. Selfridge, G. H. Martin, Augur, Holmgren, and Manning.

The minutes of September 17 were read, and, after corrections in the wording, were approved.

Dr. Manning then read a paper upon the "Second Caution of Hahnemann," giving the latter's views in the words of the former.*

This caution was directed against carelessness and laziness. It was then shown how necessary it was for a homœopath to be exact in his prescribing, and that it could only be accomplished by close study and work. So much depends upon the proper selection of the remedy. Two causes were named as proving laziness and against exactness. First, the repertory, which was spoken of as valuable if used correctly, and to be used correctly it must act as a guide to study and research, but must not be relied upon entirely. Second, the modern tablet was also mentioned as favoring carelessness and polypharmacy. Several formulas as advertised by pharmacies were mentioned, and the question asked as to how they differed from a prescription of the regular; also objection was made to them on account of their impure manufacture.

### DISCUSSION.

Dr. Selfridge—The paper hits pretty hard. I think we ought not to let it pass without putting ourselves on record

* See THE HOMŒOPATHIC PHYSICIAN for May, 1898, page 211, last line, and page 212 and following pages.

regarding the tablet. I never use them, because you cannot rely on their purity. There should be glass molds, pestles, slabs, etc., for each remedy, if we adhere to our doctrines, yet this care is not used. The molds from their shape are almost impossible to clean. Tablets are also aids to polypharmacy, as the doctor says. Moreover, they are used by allopaths to disguise their prescriptions.

Dr. Martin—I never use them.

Dr. Holmgren—A pharmacist informed me that they used acacia to cause the triturate to keep its shape, and talcum to dust the mold.

Dr. Selfridge—All such things are an injury.

Dr. Martin—Another mistake we make nowadays is in slighting Homœopathy. In earlier days if a physician made a cure he did not take the credit of the cure, it was due to Homœopathy.

Dr. Augur—So many like to have a physician who claims to practice both systems; and so many do claim it. I think the almighty dollar is at the bottom of it all.

Dr. Martin—It is our duty to train our patients. I always endeavor to train mine, and they know whether a doctor is a homœopath or a pretender.

At the close of the discussion Dr. Geo. H. Martin and Dr. Guy E. Manning were appointed essayists for the next meeting to continue the discussion of *Chronic Diseases.*

Adjourned on motion.

GUY E. MANNING,
*Secretary.*

---

The natives of a village about fifty miles from Mexico have taken a genuine Indian method of stopping the spread of smallpox, which appeared among them. The first man to take the disease was beaten to death, and they set fire to the house.

